# Effects of annealing on performances of 1.3-μm InAs-InGaAs-GaAs quantum dot electroabsorption modulators

**DOI:** 10.1186/1556-276X-8-59

**Published:** 2013-02-06

**Authors:** Shuh Ying Lee, Soon Fatt Yoon, Andrew CY Ngo, Tina Guo

**Affiliations:** 1 Division of Microelectronics, School of Electrical and Electronic Engineering, Nanyang Technological University, 50 Nanyang Avenue, S1-B2c-21, Singapore, 639798, Singapore; 2 Patterning and Fabrication Capability Group, Institute of Materials Research and Engineering, Singapore, 117602, Singapore; 3 Temasek Laboratories at NTU, Nanyang Technological University, Singapore, 639798, Singapore

**Keywords:** Quantum dot, Electroabsorption modulator, Lumped element, Annealing, Interdiffusion, 85.35.Be, 42.79.Hp, 81.40.Ef

## Abstract

In this work, we investigated the effects of quantum dot (QD) annealing (as-grown, 600°C-annealed, and 750°C-annealed) on the preliminary performances of 1.3-μm InAs-InGaAs-GaAs quantum dot electroabsorption modulators (QD-EAMs). Both extinction ratio and insertion loss were found to vary inversely with the annealing temperature. Most importantly, the 3-dB response of the 750°C-annealed lumped-element QD-EAM was found to be 1.6 GHz at zero reverse bias voltage - the lowest reverse bias voltage reported. We believe that this work will be beneficial to researchers working on on-chip integration of QD-EAMs with other devices since energy consumption will be an important consideration.

## Background

Quantum dot (QD) lasers are now extensively investigated for applications in low-cost metropolitan access and local area networks. However, most works on QD devices focus on lasers and detectors. There were only a handful of them that were related to quantum dot electroabsorption modulators (QD-EAMs) [1,2]. For ease of monolithic integration, it is timely to investigate the use of QDs for electroabsorption modulators (EAMs). As such, one can then utilize QDs for both laser and EAM by the identical active layer approach [3,4].

Recently, Chu et al. reported a small-signal frequency response of 2 GHz for the 1.3-μm QD-EAM [1]. However, the applied reverse bias was 4 V - which could lead to complications for on-chip integration since energy consumption is an issue. We had previously reported the static performance of 1.3-μm QD-EAM based on as-grown QDs [5]. Due to the defined QD potential barriers, one can observe a suppression of absorption at reverse bias <2 V [6]. This implies that our as-grown QD-EAM will also require a significant reverse bias voltage (≥2 V in this case) for small-signal frequency response. Again, this is undesirable for on-chip integration. On the other hand, annealed QDs are proposed to be a good candidate for energy-efficient QD-EAM. By varying the annealing temperature, we are able to induce different diffusion lengths on the QD layers [7]. There are two mechanisms at work, the first being the exchange of In atoms from the InAs QD intermixing with the Ga atoms in its surrounding InGaAs QW and the second being the In-Ga interdiffusion through the InGaAs/GaAs interface [8]. The uniform interdiffusion would result in a more symmetrical Stark shift and consequently reduces the built-in dipole momentum - both of which are beneficial to electroabsorption modulation. An enhanced Stark shift would theoretically provide a cleaner and larger extinction ratio, whereas the reduction of built-in dipole moment in QD would increase the ground-state electron–hole overlap. Therefore, in this work, we investigated the effect of QD annealing on the static and dynamic performances of 1.3-μm QD-EAM.

## Methods

**Figure 1:**
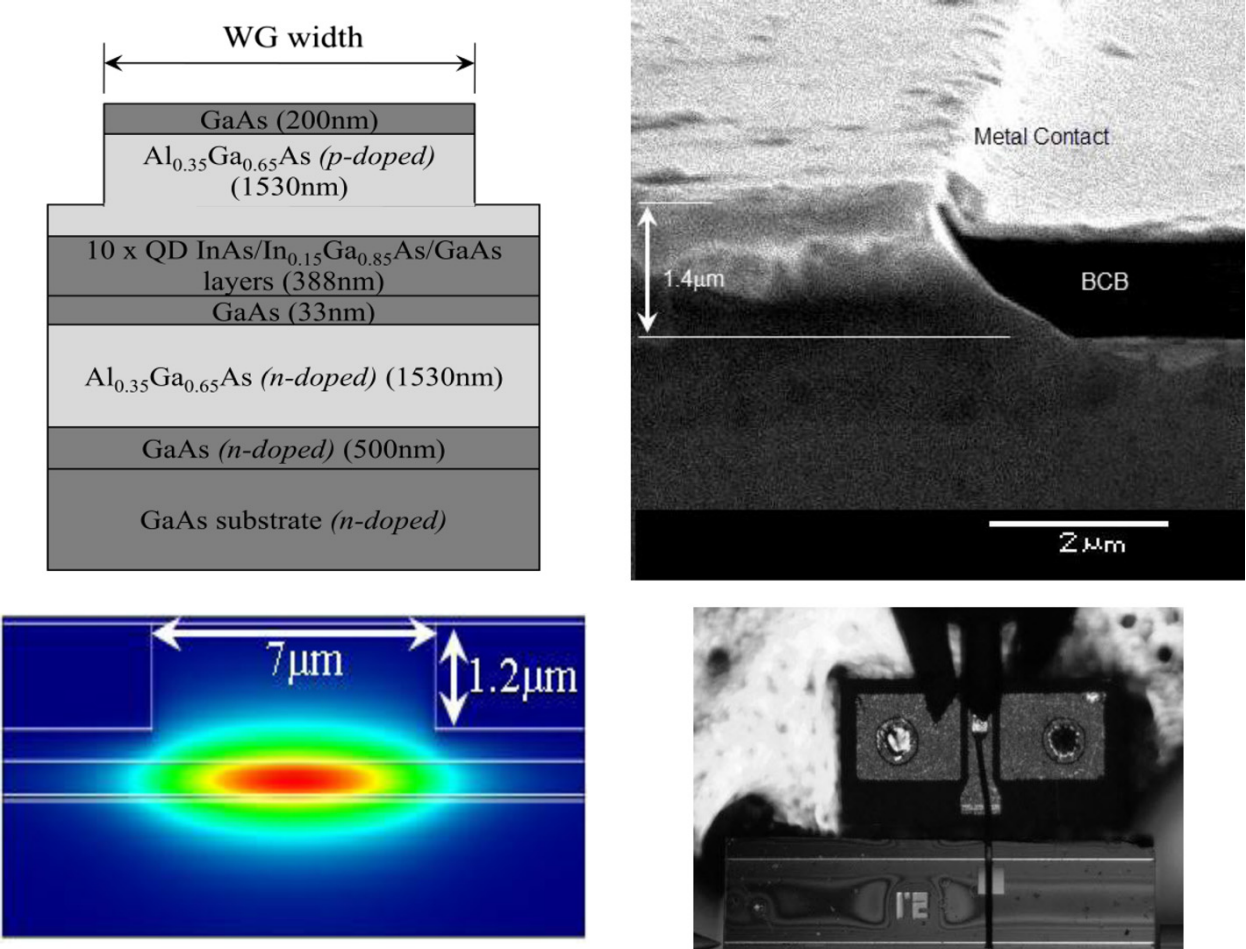
**Device fabrication.** (top left) Material structure design where WG width is 7 μm. (top right) Cross-sectional scanning electron microscopy image of a sidewall of the QD-EAM fabricated using wet-etching techniques. (bottom left) Simulation of mode formed by a 7-μm waveguide with an etch depth of 1.2 μm. (bottom right) Fabricated device which is wire bonded to a GSG pad for RF measurements.

The devices were fabricated from a QD wafer grown by molecular beam epitaxy. The undoped InAs/InGaAs/GaAs QD structure was grown on an n+−GaAs substrate as described in [6]. The laser waveguide was capped with a 200-nm GaAs n-contact layer. Figure 1 (top left) depicts the material structure of the modulator. The waveguide structures were fabricated using wet-etching fabrication techniques. The cross-sectional scanning electron microscopy (SEM) image of a sidewall of the QD-EAM fabricated using wet-etching techniques is shown in Figure 1 (top right).

The QD wafer was first deposited with 300-nm SiO_2_ using the Unaxis Neutral D2000 (St. Petersburg, FL, USA). The deposition was performed at 50°C, and the deposition rate was approximately 72 nm/min. Although the quality of the low-temperature (LT) SiO_2_ layer is not as good as that deposited at higher temperature (approximately 300°C), the quality was found to be acceptable and did not affect the refractive indices of SiO_2_[9]. It is also worth highlighting that although LT SiO_2_ is more porous and less adhesive than that deposited at higher temperature, it is sufficiently stable for our annealing needs [10]. The SiO_2_ deposition was followed by annealing at conditions which were based on previously reported works [11,12], i.e., 600°C and 750°C. Table 1 describes the annealing conditions of the QD samples under investigation. For ease of reference, they are labeled as AG, 600A, and 750A for the rest of this paper.

**Table 1:** Label and description of the QD samples under investigation

**Label**	**Description**
AG	As-grown
600A	Annealed at 600°C for 10 s
750A	Annealed at 750°C for 10 s

After the annealing process, the SiO_2_ was removed using a HF/H_2_O rinse with a ratio of 1:10. Subsequently, a photoresist was spun on the wafer surface, and the stripe patterns of the ridge waveguide (RWG) structures were defined after UV exposure and photoresist developing. This was followed by wet chemical etching (H_3_PO_4_/H_2_O_2_/DI = 1:1:5 with an etch rate of approximately 1.2 μm/min) to define the ridge height. After the wet-etching process, the remaining photoresists that acted as etching masks were removed using acetone. The next step was the planarization step. This step involved spin coating of bisbenzocyclobutene (BCB) monomers. The BCB helped to flatten out the sample surface and acted as a passivation step. The waveguide sides that had been coated with BCB also helped to reduce capacitance in high-speed measurements. The etch-back step was then applied to reduce this BCB layer until the waveguide layer was exposed again. Note that this method was preferred over the alternative method of defining photoresist pattern. This was because the RWG EAM devices had heights of approximately 1.2 μm; hence, higher chances of misalignment and poorer yield would be expected if the latter method (i.e., defining photoresist pattern) was employed. The wafer was then lapped down to approximately 100 μm before electron beam evaporation of both p-type and n-type ohmic contact layers. It is worth highlighting that the metallic p-pad, which was needed for probing or wire bonding, was designed to be as small as possible (80 × 80 μm^2^ in this case). This is because it contributed to the parasitic capacitance and was thus detrimental to the modulation bandwidth of the EAM devices. Finally, the wafer was cleaved into a ridge length of 1,700 μm (i.e., 1.7 mm) for device characterization. For higher yield and easier coupling purposes, the widths of the waveguides fabricated (WG width) were set at 7 μm. The effective index for a 7-μm-wide rib waveguide with an etch depth of 1.2 μm is approximately 3.325 and is still sufficiently narrow to hold single-mode propagation as shown in the simulation in Figure 1 (bottom left). However, careful alignment and cleaving was still necessary in order to avoid exciting higher order modes [13]. Although in actual fabrication the etch depth is 1.4 μm, 0.2 μm has been omitted in this simulation because that is for the GaAs contact layer of higher refractive index and sufficiently far away from the inserted light source that it need not be included when simulating the mode propagation. The microscopic plan view of the QD-EAM devices that were designed as basic top-bottom p-i-n elements as illustrated in Figure 1 (bottom right). The pad sizes of the devices are approximately 80 × 80 μm^2^ which is sufficiently large for probing and wire bonding purposes but small enough to avoid inducing additional capacitance to the device.

**Figure 2:**
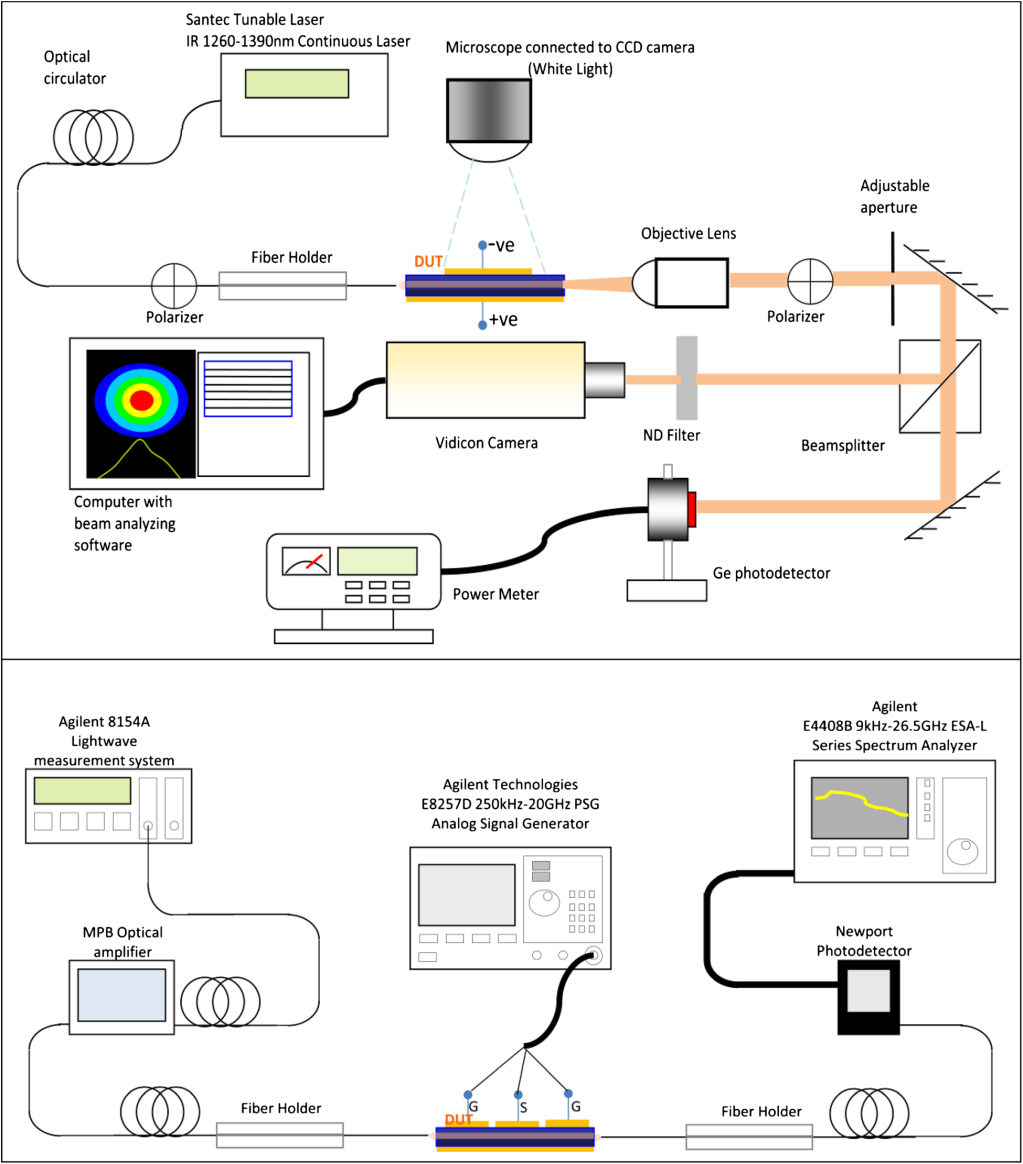
**Fiber-DUT-free space and fiber-DUT-fiber setups.** (top) Fiber-DUT-free space setup for static (DC) measurement. (bottom) Fiber-DUT-fiber setup for RF measurement.

A fiber-device under test (DUT)-free space setup as illustrated in Figure 2 (top) was used during the course of the direct current (DC) measurements for a more accurate positioning [13] and identification of the propagating mode - be it the fundamental mode or a higher order mode that was being modulated. Using an external ground-signal-ground (GSG) pad, a wire bonded to the QD-EAM, and a fiber-DUT-fiber measurement setup as illustrated in Figure 2 (bottom), we were able to perform preliminary radio-frequency (RF) measurements on the devices as shown in Figure 1 (bottom left). Since the device length is much longer than the RF wavelength, it is regarded as a lumped element, and therefore, no external impedance matching load was used. The RF signal was provided by a signal generator, and the modulated light was detected using a photodetector with known frequency response and a spectrum analyzer.

## Results and discussion

**Figure 3:**
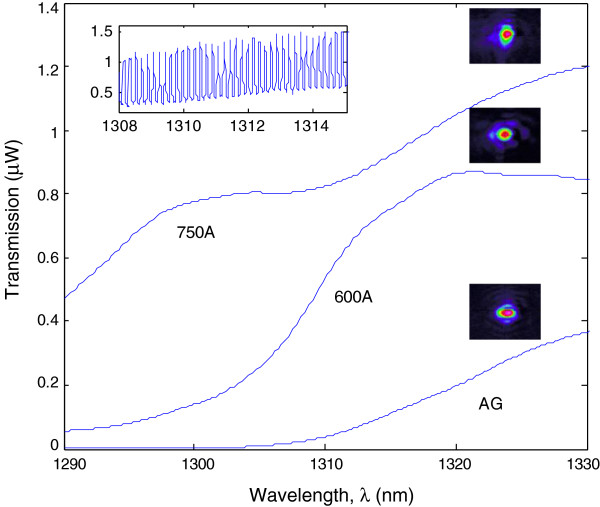
**Transmission spectra of AG, 600A, and 750A with respective single-mode shapes measured at 1,310 nm.** Inset shows the FP spectrum of 750A, which was used to calculate the propagation loss.

Figure 3 depicts the transmission spectra (1,300 to 1,330 nm) of the QD waveguide for the three types of condition measured. Note that the insertion loss of the devices improved significantly in the annealed waveguides. There was also a significant blueshift in the transmission spectrum of the 600A waveguide as compared to the AG waveguide due to the blueshift of the transition energy of QDs which is in accordance with [14].

A good indication of the single-mode propagation obtained was by observing the single-mode Fabry-Perot (FP) spectrum as shown in the inset of Figure 3. The calculated propagation loss based on the respective FP spectra was 4.0 dB/cm for AG, 3.7 dB/cm for 600A, and 3.0 dB/cm for 750A at the wavelength range of 1,308 to 1,315 nm. The improvement in the propagation loss indicated the diffusion of the QD layers and an unintentional passivation of the device. When measuring the propagation loss, shorter waveguides from the batch of devices were cleaved instead of using actual DUTs. This was because longer devices will give much finer mode spacing, and this would result in less accurate data.

**Figure 4:**
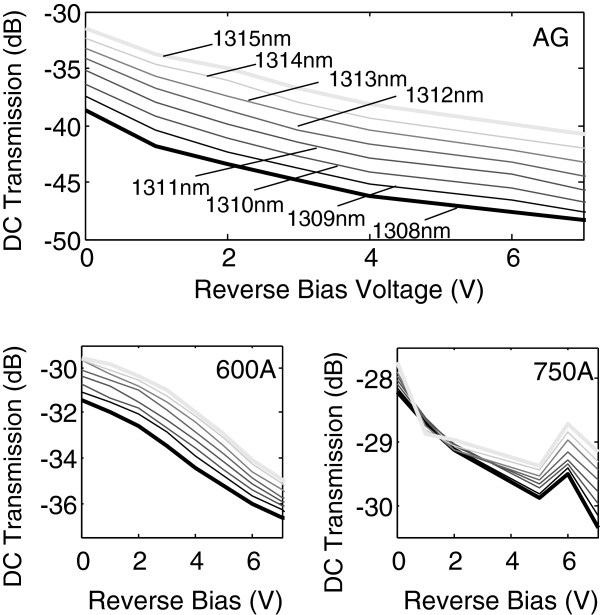
**DC transmission curves of AG, 600A, and 750A for 1,308 to 1,315 nm, in 1-nm steps.** Notice that the ‘width’ of the wavelength band decreases (hence less sensitive to wavelength change) with increasing annealing temperature.

Besides the improvement in the propagation loss, a significant change to the DUTs after annealing was that it became impervious to wavelength change, hence making the DUTs less sensitive to wavelength variation. As shown in Figure 4, when the range of transmission intensities of the AG and 600A DUTs in 1,308 to 1,315 nm were compared, an approximately 50% lesser transmission difference was observed on the latter device, i.e., the range was smaller. For example, at −4 V, the range of DC transmission was approximately 8 dB for the AG DUTs as compared to approximately 2 dB and approximately 0.5d B for DUTs 600A and 750A, respectively.

**Figure 5:**
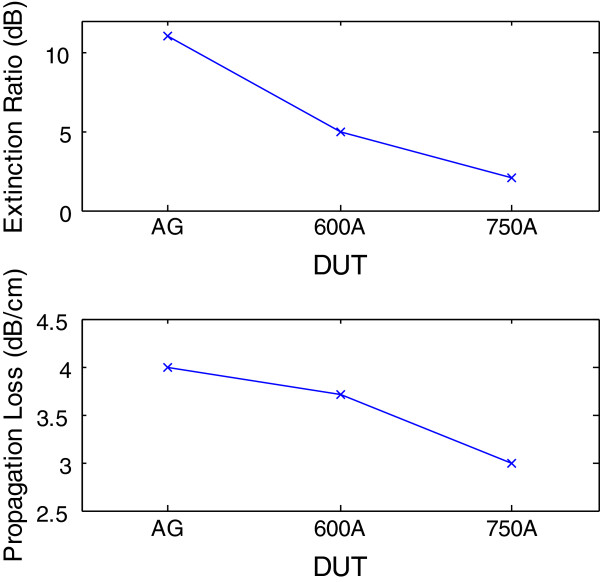
Extinction ratio (top) and propagation loss (bottom) of AG, 600A, and 750A.

The applied reverse bias voltage for the measurement of the DC optical transmission of the DUTs was capped at 7.0 V. This corresponded to the electric fields of 0 to 150 kV/cm. This was because it was too power intensive to drive an EAM at higher voltage. However, it is worth highlighting that the largest leakage current in the measured range was still less than −0.5 μA, suggesting that the breakdown voltage of the QD device was in excess of −7 V. For the as-grown DUT, we had previously reported an extinction ratio of up to 13 dB at a reverse bias of 10 V and approximately 10 dB of ON/OFF ratio for 8 V [6]. The DC measurement observed indicated that at the length of 1.6 mm, the absorption of the QD-EAM began to saturate at a reverse bias voltage of 6 V and above. Note that due to the observed suppression of absorption at low reverse bias (<2 V), a higher bias voltage was required for the as-grown device [2]. Nevertheless, since the optical power capability of conventional EAM is normally limited by the piling up of photogenerated holes as a result of heavier effective mass as compared to that of electron, a larger bias voltage would be beneficial to the power handling capability [15]. This is because the field screening effect due to the trapped holes inside the confinement region will be reduced at higher electric field [16]. In the case of the annealed samples, the intermixing lowered the field screening effect at lower electric field. Therefore, 600A demonstrated a reduced built-in potential which was in accordance with the interdiffusion induced [17]. However, the maximum extinction ratio achieved was reduced to 7 dB. The extinction ratio of 750A was further reduced to <3 dB. Hence, although interdiffusion enhances the QD Stark shifts and greatly reduced the built-in dipole moment, at a RTA range which is too high, it reduces the modulation range at higher voltage. The increased transfer curve gradient of 750A followed by weakened modulation at higher voltage could be due to the thermally induced bandgap shrinkage [18] due to the increased transmitted output light in 750A when compared to AG or 600A. The extinction ratio and propagation loss comparisons of all three DUTs are presented in Figure 5 to further illustrate the effects of annealing on these two parameters.

**Figure 6:**
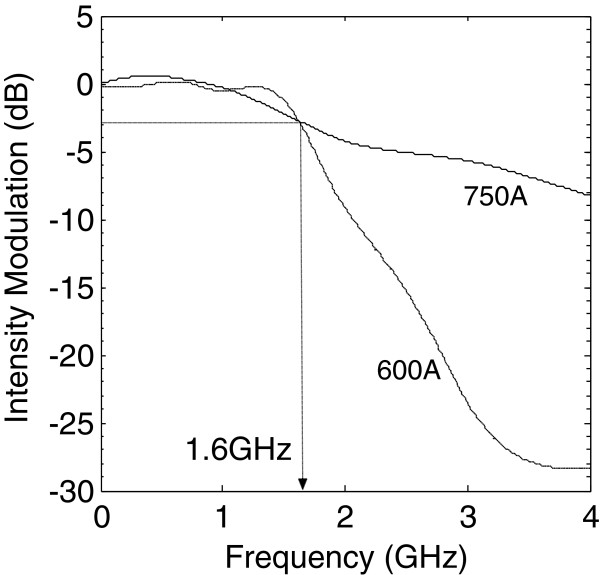
**Intensity modulation response of 600A and 750A.** Note that the reverse bias voltages are 0.5 and 0 V, respectively.

Due to the low transmitted intensity of the as-grown DUTs and limitation of the photodetector's sensitivity, only the experimental results of the annealed DUTs were obtained. Figure 6 shows the small-signal intensity modulation of the annealed DUTs measured at 1,310 nm. A significant advantage of intermixing was the reduced DC reverse bias (driving voltage) needed for the small-signal intensity modulation. A similarly structured QD EAM has been reported to demonstrate a small-signal modulation bandwidth of 2 GHz at a reverse bias of 4 V [1]. For the 600A device, the reverse bias introduced was as low as 0.5 V, and for 750A, no reverse bias was applied. The elimination of a need for DC reverse bias due to the change in the transmission curve brought on by intermixing will also reduce the complexity of the modulator setup and is a promising indication of selective annealing for on-chip integration.

Note that although the DC extinction ratio of 600A (750A) was reduced to less than 70% (30%) of its original modulation ability, RF measurement on the devices was still possible due to lower propagation loss after annealing. The 3-dB bandwidth of both 600A and 750A is approximately 1.6 GHz. Noting that these are preliminary RF results, similar frequency responses of approximately 1.6 GHz for both 600A and 750A might be due to the non-optimized WG structures and RF matching. That is, the obtained RF performance was limited by the device design and not by the QD materials. Therefore, we believe that an improvement in the high-speed performance will be expected following the optimization of QD waveguide design and improved RF matching.

The realization of RF measurement on the processed (annealed) lumped-element QD-EAM confirms the prospect of QD epiwafer in monolithic integration for future references. By applying low-cost intermixing, such integration will have low insertion loss and polarization-independent properties [14]. This is because the integrated devices would actually be made from the same epilayers unlike other types of integration. Therefore, the EAMs would naturally be tuned to the same polarization as that of the emitted radiation from the corresponding QD lasers, and improved extinction ratio may even be observed due to the improved absorption strength of the same platform that integrated devices share.

## Conclusions

In this work, we investigated the effects of annealing on the static and dynamic performances of lumped-element QD-EAM operating at the wavelength of 1.3 μm. The extinction ratio at −8 V (propagation loss) for the as-grown, 600°C, and 750°C DUTs was found to be 10 dB (4.0 dB/cm), 7 dB (3.7 dB/cm), and <3 dB (3.0 dB/cm), respectively. Hence, both the extinction ratio and the insertion loss decrease upon increase in annealing temperature. Most significantly, the 3-dB response of the 750°C-annealed lumped-element QD-EAM was found to be 1.6 GHz at *zero* reverse bias voltage. This suggests a cost- and design-effective solution to enhance transmission and will be beneficial for researchers working on the implementation of QD-EAMs in monolithic integration through the intermixing process method.

## Competing interests

The authors declare that they have no competing interests.

## Authors’ contributions

SYL carried out the electroabsorption design, fabrication, and measurements; participated in the studies of electroabsorption behavior; and drafted the manuscript. SFY conceived of the study and participated in its design and coordination. ACYN carried out the material studies and participated in the design, studies of the electroabsorption behavior, and manuscript editing. TG participated in the device measurement. All authors read and approved the final manuscript.
